# The Effect of High-Density Built Environments on Elderly Individuals’ Physical Health: A Cross-Sectional Study in Guangzhou, China

**DOI:** 10.3390/ijerph181910250

**Published:** 2021-09-29

**Authors:** Rongrong Zhang, Song Liu, Ming Li, Xiong He, Chunshan Zhou

**Affiliations:** School of Geography and Planning, Sun Yat-sen University, Guangzhou 510275, China; zhangrr5@mail2.sysu.edu.cn (R.Z.); liusong6@mail2.sysu.edu.cn (S.L.); liming57@mail2.sysu.edu.cn (M.L.); hexiong6@mail2.sysu.edu.cn (X.H.)

**Keywords:** built environment, the elderly, physical health, physical activity, social interaction activity

## Abstract

The built environment refers to the objective material environment built by humans in cities for living and production activities. Existing studies have proven that the built environment plays a significant role in human health, but little attention is paid to the elderly in this regard. At the same time, existing studies are mainly concentrated in Western developed countries, and there are few empirical studies in developing countries such as China. Based on POI (point of interest) data and 882 questionnaires collected from 20 neighborhoods in Guangzhou, we employ multilevel linear regression modeling, mediating effect modeling, to explore the path and mechanism of the impact of the built environment on elderly individuals’ physical health, especially the mediating effects of physical and social interaction activity. The results show that the number of POIs, the distance to the nearest park and square, and the number of parks and squares are significantly positively correlated with the physical health of the elderly, while the number of bus and subway stations and the distance to the nearest station are significantly negatively correlated. Secondly, physical activity and social networks play a separate role in mediating the effect of the built environment on elderly individuals’ physical health. The results enrich the research on the built environment and elderly individuals’ health in the context of high-density cities in China and provide some reference basis for actively promoting spatial intervention and cultivating a healthy aging society.

## 1. Introduction

Many countries around the world are facing severe challenges of rapid population aging [1,2], especially China [3]. China is experiencing a rapid population aging process [4], and the population of 60 years and above currently exceeds 253 million, accounting for 18.1% of China’s total population [5]; the number is expected to reach 400 million by 2050 [6]. In the US, the population aged 65 and older will double from 2000 to 2030 (from 35 to 71 million) [7]. Moreover, one in four people living in Europe and North America could be aged 65 or over [8]. According to a World Health Organization report, it is estimated that by 2050, the global population of people older than 60 will reach 2.1 billion [9], which is close to 22% of the total population [10,11].

Aging is an unavoidable process in life [1], but as people grow older, declines in physical function and problems with disease can seriously damage their health [12], which increases the pressure on the elderly in society, so improving the health of the elderly has become one of the most important social issues in the 21st century [13]. Facing the rapid growth of the global aging population, many scholars have carried out numerous studies on the factors that affect the health of the elderly and how to interfere and intervene in these factors to improve the elderly’s health outcomes [1,14,15]. Besides some factors that are closely related to health (e.g., demographic characteristics, socioeconomic status, social equity [16,17], physical exercise [18,19], lifestyle [20], diet [21]), built environment factors also play an important role in the health of the elderly [2,18,22]. Moreover, the construction or improvement of the built environment has been used as an important strategy to improve health, and it has been adopted by more and more public health projects [13].

The built environment refers to the objective material environment built by humans living in cities for life and production activities [23] and mainly includes building units (houses, schools, workplaces), open spaces (parks, squares, entertainment venues), infrastructure (transportation systems), and public service facilities (shopping malls, stadiums, libraries) [13]. With the development of global urbanization, it is estimated that by 2050, 68% of the world’s population will live in cities [24]. What will follow is that the development of the urban built environment will reduce contact between people and the natural environment [25] and increase the activities of daily life in the built environment [26,27]. Compared with younger adults, due to the low socioeconomic status [28] and mobility of the elderly [29], many elements of the built environment have not taken into account their needs [30]. At the same time, the decline in physical function brought about by aging makes them more vulnerable to the detrimental effects of physically challenging environments (e.g., inclines, residential density, mixed land use, destination accessibility, aesthetics, street connectivity, parks, and open space) on daily life [31,32], leading them to be more susceptible to the effect of the built environment [14,33]. 

In recent years, research on the relationship between the built environment and elderly individuals’ health has gradually attracted interest in the fields of urban planning, construction, and public health [34,35,36], focusing on how the built environment impacts a wide range of issues, including obesity [15,37], physical health [38], mental illness [39], and morbidity [40]. Two studies conducted in the US have investigated the linkage between neighborhood design and health outcomes and found high walkability to be associated with a healthier self-reported health status An empirical study in Belgium found that there was a significant relationship between walkability and obesity among the elderly living in low-income neighborhoods, but not with physical and mental health [41].

However, most studies focusing on the built environment and elderly’s health outcomes have mainly been conducted in Western developed countries such as the US [37,42,43] and the European countries [15,39,42,44]. Research in developing countries with high-density built environments such as China is still lacking. This may be due to the differences in the built environments in developed countries [45,46], such as population density, road density, and public transportation [47,48,49], leading to different conclusions [1,4,50]. Therefore, previous research results obtained in Western developed countries may not be applicable to developing countries.

Thus, this study aims to explore the relationship between high-density built environment and elderly individuals’ physical health in China. The results of this study will not only enrich the relevant research in the context of high-density cities in China but also provide a certain reference for active spatial intervention and the cultivation of a healthy aging society. The structure of this paper is as follows: The second part sorts out the related literature on the effect of the built environment on elderly individuals’ physical health and establishes the research framework; the third part introduces the data sources and research method; the fourth part presents the analysis of the research results; the last part is the discussion and conclusion.

## 2. Literature Review

The World Health Organization (WHO) defines physical health as the active state of physiological functions in daily life and the subjective or objective evaluation of physiological conditions. 

Bronfenbrenner [51] introduced ecological theory into social science and urban science research, emphasizing the importance of the impact of the environment on human health. The theory holds that human health is not only affected by individual physiological characteristics (e.g., genetics, development status), lifestyle, and other factors but is also closely related to urban land use, building density, transportation systems, and other built environment factors [52]. Cervero and Kockelman [53] put forward a three-dimensional evaluation of the built environment from the aspects of density, mixing degree, and design. Then, based on 3D, Ewing and Cervero [54] added two dimensions, destination accessibility and distance to public transport, forming a five-dimensional evaluation of the built environment. At present, many studies are based on the 5D evaluation, selecting indicators such as population density [23,55,56], land use [57,58], green space (parks) [59,60], destination accessibility [56,58], and distance to public transport [56,61] to explore the relationship between the built environment and residents’ health [27,45]. Other studies have examined the built environment and specific physiological and psychological diseases (e.g., coronary heart disease, hypertension, type 2 diabetes, depression) [62,63].

Since then, scholars have emphasized the importance of the built environment to elderly individuals’ health [30,64]. For example, Putrik, van Amelsvoort [15] investigated 9771 elderly people in Maastricht, the Netherlands, and found that the built environment had a significant positive impact on their health. Older people living in neighborhoods with a more walkable environment (e.g., better facility accessibility, open spaces such as parks and green spaces, and road safety) are usually more active and in better health [38,43,65]. However, a study from Canada conducted the 12 years of follow-up of the elderly group and found that walkability had a greater impact on men’s weight than women’s. Moving to a neighborhood with low walkability increased the BMI for men by approximately 0.45 kg/m^2^, while women’s weight did not change significantly [63]. Therefore, so far, there is no clear research conclusion on this relationship.

The path between the built environment and individual health is complex [66], with not only direct effects but also indirect effects through the mediating path. Existing studies mostly explore the mediating role of physical activity. Insufficient physical activity has been identified as the fourth-highest risk factor leading to death globally, causing about 3.2 million deaths every year [67]. Active participation in physical activity, even moderate physical activity, can have a significant positive effect on maintaining good physical condition [68]. Many studies have been done on the mediating effect of physical activity, showing that the built environment can reduce the probability of occurrence of physiological diseases such as obesity, hypertension, and diabetes by promoting physical activity, thereby affecting people’s health [19,62]. For example, scholars have found that in the context of low population density in Western developed countries, increased population density shortens the distance between daily travel destinations and can promote transportation modes such as walking and cycling so as to increase physical activity, reduce obesity, and improve physical health [57]. Evidence from California also showed that residents in neighborhoods with higher density, land use mix, street connectivity, and safety spent more time in physical activity and had lower obesity prevalence [69]. A recent before and after study of downtown Vancouver demonstrated that opening urban greenways and reallocating road space from motor vehicles to other activities can increase active travel and physical activity time so as to improve health conditions [70]. However, the research also showed that while high-density neighborhoods can encourage active transportation, neighborhoods that have high population density also have a higher level of economic development. In areas with high economic development, people usually walk less and have more opportunities to consume unhealthy food, and the living environment is also noisier, which can increase the risk of obesity and mental illness [71]. 

Similarly, land use diversity also has a positive or negative impact on health through physical activity [47,56]. Parks and squares provide residents with space and opportunities for physical activity and promote healthy behavior [72]. Diez Roux, Evenson [73] conducted research on New York City and Forsyth County to investigate whether the availability of recreational resources is related to physical activity levels and found that adults living in areas with a large number of entertainment spaces and high park density were more likely to engage in physical activity. Additionally, physical activity has special significance and benefits for the physical and mental health of the elderly, which has also been confirmed in an empirical study in three towns: Amsterdam, Doetinchem, and Maastricht [74]. However, some studies have found that the presence of parks and squares may negatively affect health [75]. Although the mediating role of physical activity in the relationship between the built environment and health has been fully studied [19,61,76], there has been relatively little attention paid to the elderly. Studies on the elderly often pay more attention to the impact of the characteristics of the built environment on physical activity [41] or specific health indicators, such as obesity, overweight, or chronic diseases [77,78]. For example, Creatore, Glazier [62] carried out a study in Ontario, Canada, and demonstrated that the incidence rate of diabetes in elderly people who engage in walking and leisure sports activities is decreasing. Other studies have found that accessibility to destinations such as parks, open spaces, and playgrounds and walkability features such as neighborhood safety and covered sidewalks can promote the use of transportation and outdoor recreational sports activities by elderly residents [66].

In addition, social interaction activities also represent an important mediating path that can affect health by providing social support and expanding social networks [79]. In Canada, Pearce and Kristjansson [80] reported a positive correlation between the scale of social networks and the availability of built-up environmental factors (such as parks and services), indicating that the scale of social networks may increase with the improvement of the availability of built up environmental factors in neighborhoods. The increase in the scale of social networks improves the possibility of informal meetings between the elderly [81,82] and meets the daily social needs of the brain, which plays a significant role in improving health outcomes [83]. Moreover, diversified land use can shorten the distance between places for leisure activities and increase people’s social interaction opportunities [84], which helps to reduce the risk of obesity and chronic diseases and improve physical health [85]. However, some studies have found that diversified land use makes the neighborhood more noisy and crowded, canceling out the positive effects on health [86]. The distance to parks and squares has been found to improve health by increasing social interaction opportunities for residents and improving the level of social interaction [72], but some studies have found that the presence of parks and squares may negatively affect health [75]. The distance to bus stops can promote the improvement of physical activity [87] and social interaction [88] by increasing the chance that people will choose public transportation, thus reducing the risk of obesity and other chronic diseases [56,61], and can also help to improve the level of physical health. However, some studies have found that there is a significant negative correlation between the proximity of bus stops and the level of physical health. This may be because the closer the distance to the bus stop, the less time people will walk or ride and the less physical activity they will have. Therefore, the dependence on public transport and the reduction in social interaction activity will have an adverse impact on people’s health [89]. In addition, Cabrera and Najarian [90] found that residents in Tucson, Arizona, living near the main roads and bus stations were disturbed by the heavy traffic and were less likely to know their neighbors and make new friends.

Although many empirical studies have proven that social interaction activity constitutes a mediating path and achieved rich research results on the relationship between the built environment and physical health, there are few such empirical studies focused on the elderly. For example, a study by Beard, Blaney [91] found that reduced walkability of the neighborhood environment could reduce social activities, resulting in reduced activity, decreased physical function, and reduced social support for the elderly, which can result in increased loneliness, which is not conducive to health. Other studies have investigated the relationship between the green space in the neighborhood and the social interaction activity of the elderly and found that social interaction activity is affected by trees, the availability of grass, and the greenness of the space and its safety and maintenance [92,93].

**Figure 1:**
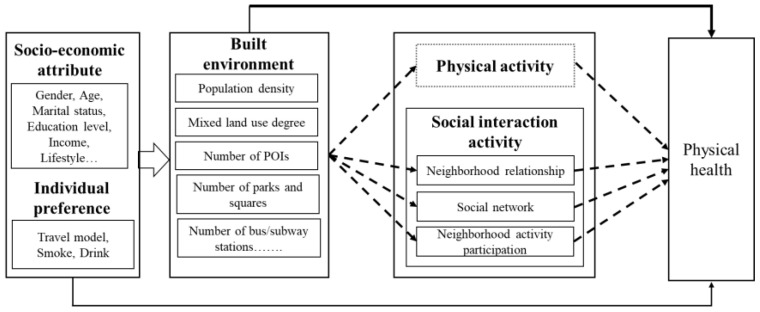
Research framework.

Based on the above literature review, we find that the research on the relationship between the built environment and elderly individuals’ physical health is mainly concentrated in developed countries, and there is no unified understanding of this relationship. In addition, the mediating path of physical activity and social interaction activity in this relationship is ignored. In light of these research gaps, this study aims to evaluate the relationship between the built environment and the health of the elderly by examining 882 elderly people from 20 neighborhoods in Guangzhou based on questionnaire survey data, POI data, and other data sources (e.g., sixth and seventh national population census in China). In particular, it focuses on the extent to which physical and social activity regulate the link between the built environment and elderly individuals’ physical health (Figure 1). This study expands previous research in two aspects. First, by revealing the potential mechanism, it increases our understanding of the health of the elderly in the context of high-density cities in China in order to make a comparison with other low-density developed countries. Second, a multilevel mediating effect model is used to quantify the direct and indirect effects of the built environment on the physical health of the elderly.

## 3. Data Sources and Methods

### 3.1. Data Sources

In Guangzhou, one of China’s megacities, a rapid change of urban spatial form has also brought about changes in the built environment [94]. Meanwhile, the elderly population in Guangzhou has also increased rapidly. By the end of 2019, 1,755,100 people aged 60 and over resided in the city, accounting for 18.40% of the registered population. From December 2018 to April 2019, we conducted a questionnaire survey of elderly persons over 60 years old who had been living in Guangzhou for more than 6 months.

**Figure 2:**
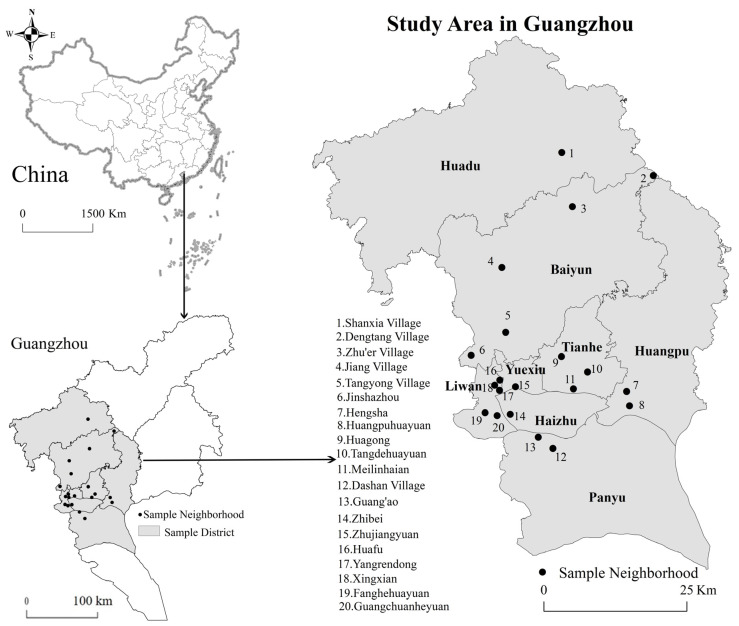
Location of sample neighborhoods.

A multistage stratified probability proportionate to population size sampling technique (PPS) was adopted to extract samples. PPS is a probability sampling proportional to the size of the scale. It is often used in two-stage sampling. In the first stage, according to the sixth national population census in China, we extracted the original data reflecting the social attributes of the aging population in Guangzhou, such as demographic characteristics, socioeconomic characteristics, and housing conditions, divided the social aging areas in Guangzhou into six types using ecological factor analysis and cluster analysis (including concentrated distribution areas of older adults in old neighborhoods, in government agencies, enterprises, and institutions, in urban villages, and in new development areas of the younger generation, and scattered distribution areas of the retired elderly in education and scientific research units, and mixed population distribution areas). Then, 18 subdistricts with the highest scores of main relevant factors among these six social aging areas were selected. Next, 20 neighborhoods with more than 10% of the elderly within these 18 subdistricts, covering six housing types, including historical neighborhoods, Danwei neighborhoods, urban villages, commercial housing, affordable housing, and rural villages, which were located in central (including Liwan, Yuexiu, and Haizhu), transitional (including Tianhe, Baiyun, and Huangpu), and marginal (including Panyu and Huadu) districts of Guangzhou (Figure 2) were selected.

In the second stage, the number of questionnaires in each neighborhood was based on the proportion of the elderly population. Respondents from each sampled neighborhood were randomly selected. Each questionnaire was administered by a trained interviewer in a face-to-face interview with a participant. Finally, out of a total of 1000 questionnaires, 974 valid questionnaires were returned, and 882 questionnaires were used to analyze the data after eliminating some invalid questionnaires (Table 1).

The questionnaire contained two parts. The first part was about the respondents’ demographic information. The second part was about the construct items designed to test the degree of the respondent’s agreement with the items, which have been placed in the Appendix A at the end of this paper. In addition to the questionnaire data, we also used the seventh national population census in China and POI data in 2019 to construct the built environment indicators of the neighborhood. 

### 3.2. Variables and Measurement

#### 3.2.1. Dependent Variables

To measure physical health, we employed the MOS 36-Item Short-Form Health Survey (SF-36) [95], which is commonly used in the health literature [96]. Physical health consists of three items that are related to self-evaluation of physical health, physical function, and physical pain. Each item is rated on a five-point Likert scale [33]. Self-evaluation of physical health was rated on a scale of 1–5 as very poor, poor, average, good, and very good. Physical function is evaluated with the question, “Are there restrictions on activities with a large amount of exercise?”. Physical pain is evaluated with the question, “Have you had physical pain in the past 4 weeks?”. Based on the Likert scale, each question had five options, scored from 1 to 5: strongly agree, agree, generally agree, disagree, and strongly disagree. The dependent variable can be treated as a continuous variable, since it was basically normally distributed [97], following prior studies [96,97]. Then, we chose the Likert scoring method to calculate the score of physical health since it contained the most information for the linear regression model (Cronbach’s alpha was 0.912 in this study, indicating good internal consistency). The Likert scoring method is scored by adding items of the same construct [4,96]. The total score of physical health was generated by adding three items ranging from 3 to 15. Higher scores indicate better physical health [96].

#### 3.2.2. Independent Variables

Compared with younger individuals, the built environment characteristics of neighborhoods are more important to the elderly since they spend most of their time at home and in the neighborhood after retirement [98]. The built environment indicators in this study are derived using 1 km buffers on Euclidean (straight-line) distance from the location of each elderly individual’s neighborhood committee, following prior studies [96,99,100]. Moreover, numerous studies have explored the relationship between the built environment and health based on the “5D” [15,45,57,67], namely, density, diversity, design, destination accessibility, and distance to destination [54]. Based on previous research [23,56,58,59,60,61] and the availability of data, seven variables were selected to measure the characteristics of the built environment. Definitions of these variables are provided as follows.

(i) Population density: Population density is defined by population divided by the sub-district area.
(ii) Land use mix: The concept of information entropy is introduced to calculate the land use mix. This principle was originally applied as a method to measure energy conservation in physics [101,102]. According to the principle of thermodynamic conservation, more intense interaction between molecules means a higher entropy of the system. The model was designed as follows:
  (1) $$H\left(x\right)\;=\;-\sum_{i=1}^{n}P_{i}logP_{i}$$
  where *H(x)* is the entropy of neighborhood *x*; *P_i_* is the probability of the appearance of different types of POIs within the 1 km buffer based on a participant’s neighborhood committee location. Obviously, the higher the entropy, the higher the land use mix.

(iii) Accessibility: We used the number of public facilities within the 1 km buffer as the proxy for accessibility, including the number of POIs, the number of parks and squares, and the number of bus and subway stations.
(iv) Distance to the destination: We used the distance to the nearest public facilities within the 1 km buffer as the proxy for distance to the destination, including distance to the nearest bus or subway station and distance to the nearest park or square.

#### 3.2.3. Control Variables

To eliminate the influence of self-selection, we took the individual socioeconomic attributes and individual preferences of the elderly as control variables. The social–economic attributes included age, gender, marital status, education level, income, lifestyle, and individual preferences, including travel model, smoking, and drinking.

#### 3.2.4. Mediating Variable

The mediating variables of this study include physical and social interaction activity. The main question regarding physical activities was, “How long do you exercise in a day (including walking)?”. Social interaction activity includes neighborhood relationships, social networks, and neighborhood activity participation. The main questions were, “Do you think the relationships in the neighborhood are harmonious?”, “Do you know many people in the neighborhood?”, and “Do you often participate in neighborhood or park activities?”. Based on the Likert scale, each question had five options, scored from 1 to 5: strongly disagree, disagree, generally agree, agree, and strongly agree.

### 3.3. Method

The physical health of the elderly in this paper is a continuous variable. Since the multilevel linear regression model can fully consider the nesting of data and accurately calculate the contributions of elements at different geographical levels, the multilevel intermediary model, which combines the multilevel linear regression model and the intermediary effect, was adopted. In this nested data structure, individuals are nested in neighborhoods. Specifically, the elderly in the same neighborhood have different socioeconomic attributes and health characteristics, but the objective built environment of the neighborhood is the same for the elderly of the same neighborhood. The differences in the physical health of the elderly in different neighborhoods are partly caused by the differences in the built environment of different neighborhoods.

**Figure 3:**
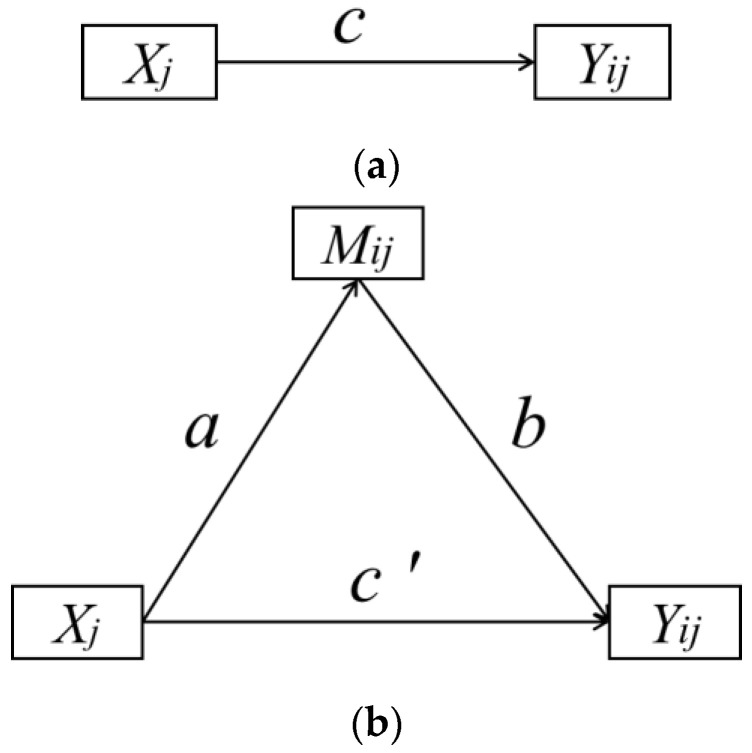
Multilevel mediating effect model. Note: *Y_ij_* represents the health of elder *i* in neighborhood *j*, which is a dependent variable; *X_j_* represents the built environment of neighborhood *j*, which is an independent variable; *M_ij_* represents the physical activity, neighborhood relationship, social network, and neighborhood activity participation of elder *i* in neighborhood *j*, which is the mediating variable.

Multistep mediation analysis was used to decompose the effect of the built environment on physical health into a direct and mediating component as well as a total effect [103] (Figure 3). Figure 3a shows the path of the independent variable (*X_j_*) to the dependent variable (*Y_ij_*); the path coefficient is *c* (total effect of *X_j_* on *Y_ij_*). Figure 3b shows the relationship between the independent variable (*X_j_*) and the dependent variable (*Y_ij_*) after controlling the mediating variable (*M_ij_*), in which coefficient *a* represents the effect of the independent variable (*X_j_*) on the mediating variable (*M_ij_*), and coefficient *b* represents the effect of the mediating variable (*M_ij_*) on the dependent variable (*Y_ij_*). *a* × *b* is the mediating effect between the independent variable (*X_j_*) and the dependent variable (*Y_ij_*) when *a*∗*b* passes the statistical significance test. Additionally, the key of the multistep mediation analysis is to test the statistical significance of the mediating effect (*a*∗*b*). Coefficient c’ is the direct effect of the independent variable (*X_j_*) on the dependent variable (*Y_ij_*); The total effect of the independent variable on the dependent variable is equal to the direct effect plus the mediating effect (*c* = *c’* + *a* × *b*).

The analysis involved the following. First, we regressed physical health on the built environment and covariates (Model 1) and obtained coefficient *c*. Second, we regressed four mediators on the built environment and covariates (Model 2a–2d) to identify the effect of the built environment on the mediators and obtained coefficient *a*. Third, we regressed physical health on the built environment, four mediators, and covariates (Model 3a–3d) and obtained coefficient *c’* and *b*. Finally, we used a bootstrap test to test the statistical significance of the mediating effect. The formula of the regression model at each stage is as follows:

Model 1:

(2) $$Y_{ij}=cX_{j}+\beta_{1}W_{ij}+\gamma_{1}Z_{j}+\alpha_{1}+\mu_{1j}+\varepsilon_{1i}$$

Model 2a–2d:

(3) $$M_{ij}=aX_{j}+\beta_{2}W_{ij}+\gamma_{2}Z_{j}+\alpha_{2}+\mu_{2j}+\varepsilon_{2i}$$

Model 3a–3d:

(4) $$Y_{ij}=c\prime X_{j}+bM_{ij}+\beta_{3}W_{ij}+\gamma_{3}Z_{ij}+\alpha_{3}+\mu_{3j}+\varepsilon_{3i}$$

where *Y_ij_* represents the physical health of elder *i* in neighborhood *j*; *X_j_* represents the built environment of neighborhood *j*; *W_ij_* represents the individual-level variable of elder *i* in neighborhood *j*; *M_ij_* represents the physical activity, neighborhood relationship, social network, and neighborhood activity participation of elder *i* in neighborhood *j*; and *Z_j_* represents the neighborhood scale variable of neighborhood *j*. *β*_1_, *β*_2_, *β*_3_ and *γ*_1_, *γ*_2_, *γ*_3_ are the variation coefficients of the individual-level and neighborhood-level variables, respectively; *μ*_1*j*_, *μ*_2*j*_, *μ*_3*j*_ represent the random effects of unobservable factors at the neighborhood scale; *ε*_1*i*_, *ε*_2*i*_, *ε*_3*i*_ represent the random effects of unobservable factors at the individual scale; and *α*_1_, *α*_2_, *α*_3_ are constants.

## 4. Results

### 4.1. Descriptive Statistical Analysis of Samples

A descriptive statistical analysis of the samples is shown in Table 2. The elderly in this survey were mainly 60–75 years old, accounting for 79.205% of the total sample. The proportions of men and women were almost the same, with the proportion of women (56.364%) slightly higher than that of men (43.636%). The education levels were mainly primary school and below (40.795%), junior middle school (27.614%), and senior high school or technical secondary school (24.886%). The per capita monthly income of the elderly was RMB 4739.13. The proportions of those living with a spouse or alone (49.659%) and living with children (50.341%) accounted for about half each. They tended to walk or ride (72.841%), and fewer people smoked (17.841%) or drank (9.205%). Their daily physical exercise duration was about 1.5 h, their neighborhood relationship (3.969) and social networks (3.732) were in good condition, and they participated in neighborhood activities to a lesser degree (only 1.720). Their average physical health score was 10.454.

The average population density of the surveyed neighborhood was 1.944, and the average land use mix was 0.667. The average number of public facilities within the 1 km buffer zone around the neighborhood was 4044.568, the number of parks and squares was 4.734, the number of bus and subway stations was 28.733, and the distance to the nearest park or square was about 500 m. The distance to the nearest bus or the subway station was about 300 m.

### 4.2. Relationship between Built Environment and Elderly Individuals’ Physical Health

Before regression analysis, it is necessary to test the feasibility of the multilevel regression model, which needs to be completed by calculating the intraclass correlation coefficient (ICC) of the null model (without any variable) [33,104]. In our study, the ICC calculated by Stata 12.0 software was 0.134 (ICC > 0.06), indicating that the inter-neighborhood difference on the physical health level explains 13.44% of the overall difference. The application of multilevel models was justifiable.

Then, Stata 12.0 software was used to analyze the association between the built environment and the elderly’s physical health (Table 3).

Model 1 is the benchmark model, which only has the built environment and individual socioeconomic attributes. The results show that in terms of the built environment, the number of POIs (β = 0.0002322, *p* < 0.05), distance to the nearest park and square (β = 0.578, *p* < 0.05), and the number of parks and squares (β = 0.060, *p* < 0.1) are positively correlated with the physical health of the elderly. The number of bus and subway stations (β = −0.032, *p* < 0.05) and distance to the nearest bus or subway station (β = −1.833, *p* < 0.05) are negatively correlated with the physical health of the elderly. No significant correlation was found between population density, land use mix, and the physical health of the elderly. 

In terms of individual socioeconomic attributes, the results show a significant negative correlation between the age and physical health of the elderly (β = −0.516, *p* < 0.05), whereas gender (β = 0.327, *p* < 0.1), education level (β = 0.507, *p* < 0.05), and income (β = 0.322, *p* < 0.05) are significantly positively correlated.

### 4.3. Relationship between Built Environment and Possible Mediators

Models 2a–d further take physical activity, neighborhood relationship, social network, and neighborhood activity participation as dependent variables, respectively, to test the relationship between the built environment and mediating variables (Table 3). 

In Model 2a, the number of parks and squares is significantly positively correlated with physical activity (β = 2.766, *p* < 0.1) and age (β = −14.873, *p* < 0.05), and income (β = −4.930, *p* < 0.1) is significantly negatively correlated with physical activity; in contrast, education level is significantly positively related with the physical activity of the elderly. 

In Model 2b, the distance to the nearest bus and subway stations is significantly negatively correlated with neighborhood relationships (β = −0.559, *p* < 0.05); education level (β = −0.274, *p* < 0.1) is significant negatively correlated with neighborhood relationships, while income (β = 0.051, *p* < 0.05) is significant positively correlated with neighborhood relationships.

In Model 2c, the number of bus and subway stations (β = −0.018, *p* < 0.05) and distance to the nearest bus and subway stations (β = −1.586, *p* < 0.05) are significantly negatively correlated with social networks; gender (β = −0.149, *p* < 0.05) is significant negatively correlated with social networks while income (β = 0.191, *p* < 0.001) is significant positively correlated with social networks. Additionally, older adults who take public transport (β = 0.348, *p* < 0.05) have larger networks.

In Model 2d, population density (β = −0.069, *p* < 0.05) and distance to the nearest bus or subway stations (β = −0.650, *p* < 0.05) are significantly negatively correlated with neighborhood activity participation; the number of parks and squares (β = 0.019, *p* < 0.05) and distance to the nearest parks and squares (β = 0.230, *p* < 0.05) are significantly positively correlated with neighborhood activity participation. Moreover, older adults who have more money (β = 0.047, *p* < 0.1) and drive or take taxis (β = 0.123, *p* < 0.05) are more likely to join in neighborhood activities.

### 4.4. Relationship between Built Environment, Mediators, and Elderly Individuals’ Physical Health 

We then added mediating variables to the benchmark model for regression analysis to verify whether they have mediating effects on the physical health of the elderly (Models 3a–d) (Table 4).

We found that the number of POIs in Model 3a (β = 0.0002253, *p* < 0.05) and the distance to the nearest park and square (β = 0.581, *p* < 0.05) are significantly positively correlated with physical health and the number of parks and squares (β = 0.056, *p* < 0.1). The number of bus and subway stations (β = −0.034, *p* < 0.05) and distance to the nearest bus and subway stations (β = −1.909, *p* < 0.05) have a significant negative correlation with physical health, and physical activity (β = 0.401, *p* < 0.05) is positively correlated with the physical health of the elderly. Age (β = −0.456, *p* < 0.05) is significantly negatively correlated with physical health, while education level (β = 0.453, *p* < 0.1) and income (β = 0.340, *p* < 0.001) are significantly positively correlated with physical health.

The number of POIs in Model 3b (β = 0.0002389, *p* < 0.05) and the distance to the nearest parks and squares (β = 0.557, *p* < 0.05) are significantly positively correlated with the physical health of the elderly, and the number of parks and squares (β = −0.060, *p* < 0.05), the number of bus and subway stations (β = −0.031, *p* < 0.05), and distance to the nearest bus or subway station (β = −1.668, *p* < 0.05) are negatively correlated with the physical health of the elderly. Age (β = −0.497, *p* < 0.05) is significantly negatively correlated with physical health, while education level (β = 0.494, *p* < 0.05) and income (β = 0.307, *p* < 0.05) are significantly positively correlated with physical health.

The number of POIs in Model 3c (β = 0.0002429, *p* < 0.05) and the distance to the nearest parks and squares (β = 0.528, *p* < 0.1) are significantly positively correlated with the physical health of the elderly; the number of parks and squares (β = −0.069, *p* < 0.05), the number of bus and subway stations (β = −0.028, *p* < 0.05), and distance to the nearest bus and subway stations (β = −1.507, *p* < 0.05) have a significant negative correlation with the physical health of the elderly; social networks (β = 0.216, *p* < 0.05) are positively correlated with the physical health of the elderly. Age (β = −0.494, *p* < 0.05) is significantly negatively correlated with physical health, while education level (β = 0.508, *p* < 0.05) and income (β = 0.279, *p* < 0.05) are significantly positively correlated with physical health.

The number of POIs in Model 3d (β = 0.00023, *p* < 0.05) and the distance to the nearest park and square (β = 0.524, *p* < 0.1) are significantly positively correlated with the physical health of the elderly; the number of parks and squares (β = −0.065, *p* < 0.05), number of bus and subway stations (β = −0.033, *p* < 0.05), and distance to the nearest bus and subway stations (β = −1.682, *p* < 0.05) have a significant negative correlation with the physical health of the elderly; neighborhood activity participation (β = 0.233, *p* < 0.05) is positively correlated with the physical health of the elderly. Age (β = −0.496, *p* < 0.05) is significantly negatively correlated with physical health, while education level (β = 0.497, *p* < 0.05) and income (β = 0.331, *p* < 0.05) are significantly positively correlated with physical health.

In order to test whether the mediating effect of the above variables is significant, the bootstrap test was conducted. According to the 95% confidence interval in Table 5, physical activity plays a significant mediating role in the path of the number of parks and squares. Further analysis shows that the number of parks and squares (β = 2.766, *p* > 0.1), the coefficient in Model 2a, is not significant; physical activity (β = 0.004, *p* < 0.05) and the number of parks and squares (β = 0.581, *p* < 0.05), the coefficients in Model 3a, is significant. The sign obtained by multiplying these two coefficients is positive, which is the same as the sign of the coefficient of the number of parks and squares in Model 3a; hence, physical activity plays a partial mediating role.

Neighborhood relationships and social networks play significant mediating roles in the path of the number of POIs. Further analysis shows that the number of POIs (β = −0.00004, *p* > 0.1), the coefficient in Model 2b, is not significant. The neighborhood relationships (β = 0.296, *p* < 0.05) and the number of POIs (β = 0.0002, *p* < 0.05), the coefficient in Model 3b, are significant, and the sign obtained by multiplying these two coefficients is negative, which is the opposite sign of the coefficient of the number of POIs in Model 3b; hence, neighborhood relationships have a suppression or inconsistent mediation effect. Similarly, social networks also have a suppression or inconsistent mediation effect on the path of the number of POIs.

The social network plays a significant mediating role in the path between the number of bus and subway stations and the distance to the nearest station. Further analysis shows that the number of bus and subway stations (β = −0.018, *p* < 0.05), the coefficient in Model 2c, is significant. Social networks (β = 0.216, *p* < 0.05) and the number of bus and subway stations (β = −0.028, *p* < 0.05), the coefficient in Model 3c, are significant, and the sign obtained by multiplying these two coefficients is negative, which is the opposite sign of the coefficient of the number of bus and subway stations in model 3c. Therefore, social networks play a partial mediating role in the path of the number of bus and subway stations. Similarly, social networks play a partial mediating role in the distance to the nearest bus and subway station.

## 5. Discussion

Consistent with the existing studies on developed countries, our results confirmed the significant relationship between China’s high-density urban built environment and the health of the elderly [41,44,45]. Specifically, the number of POIs, the distance to the nearest park and square, and the number of parks and squares were shown to be significantly positively correlated with elderly individuals’ physical health.

With the development of urbanization in China, urban space has experienced a rapid expansion stage, followed by the construction of infrastructure and public service facilities, which provide convenient living conditions for residents. For the elderly, due to the influence of Chinese traditional culture, most of them will assume the role of looking after and picking up their grandchildren, buying vegetables, cooking, and so on. The more POIs there are around their neighborhoods, the shorter the daily shopping and travel distance, which will encourage more elderly people to walk or ride to supermarkets, schools, and shopping malls, increasing their physical activity, which is conducive to improving their health [56,57,85]. Moreover, among facilities for daily activity, medical service facilities play a more positive role in the physical health of the elderly [105,106,107]. In addition, the distance to the nearest park and square and the number of parks and squares enable the elderly to engage in sports activities and healthy behavior. Furthermore, parks and squares provide social activity spaces for the elderly, which can expand their interactions and social networks and contribute to their physical health [50,66,72].

The number of bus and subway stations and the distance to the nearest station are significantly negatively correlated with the physical health of the elderly. The high-density road network structure in Chinese cities not only makes travel convenient but also makes it easy for elderly people (who used to walk and ride) to be more dependent on public transport, reducing their level of physical activity, increasing the risk of obesity and overweight, and adversely affecting their health [89]. In addition, in the context of China, fast food restaurants are often arranged around bus and subway stations, which increases the possibility that elderly people in neighborhoods close to the stations will eat out, which may increase the risk of obesity [47] and is not conducive to their physical health. This is different from some research conclusions in Western developed countries because, in the low-density built environment in those countries, people’s transportation mode is dominated by cars. Increased bus and subway station density increases the possibility that people will choose public transportation [44], thus increasing their physical and social interaction activities and improving their physical health [87,108].

In addition, our results also confirm the mediating pathway of physical and social interaction activities between the built environment and the physical health of elderly individuals [42,88,89]. Physical activity has a partial mediating effect between the number of parks and squares and physical health. Social networks have a partial mediating effect between the number of bus and subway stations and the distance to the nearest station and physical health.

**Figure 4:**
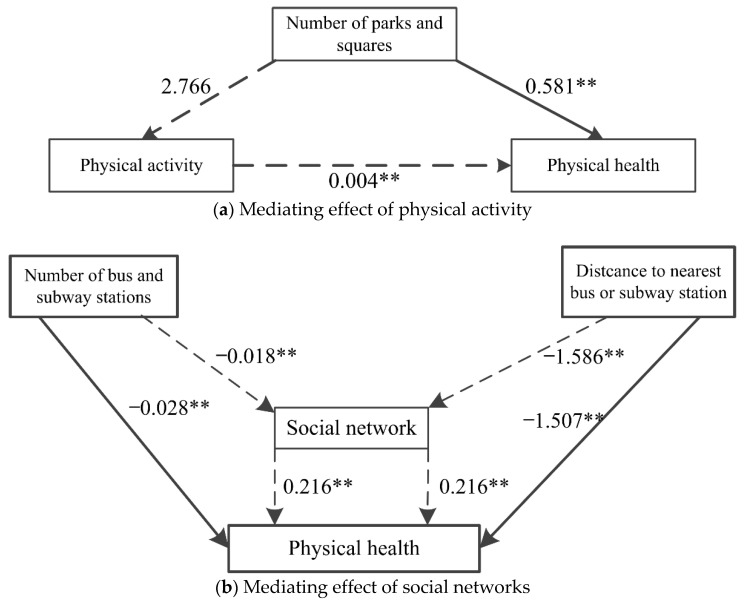
Path of mediating effect. Note: ** represents significant at 5% threshold level.

Previous studies in Western developed countries have shown that parks and squares can improve the health of the elderly by increasing the space and opportunity for physical activities [65,72]. However, in the built environment of high-density cities in China, this path is rarely explored. Consistent with the results found in the literature, we found that the number of parks and squares around the neighborhood has a positive effect on promoting physical activity for the physical health of the elderly (Figure 4a). This finding confirms the hypothesis that parks and squares provide space and opportunities for the elderly to engage in physical activities, promote healthy behavior, and provide a strong guarantee of physical health [72,109]. The elderly in Guangzhou are no exception, mainly because, compared with other age groups, they have weak mobility and relatively small space for activity. Therefore, open spaces in parks and squares are particularly important for the elderly [109,110]. At the same time, we also found that exercise facilities are often distributed in or around parks and squares, and these facilities also provide good opportunities for the elderly to engage in physical activities. 

In terms of social interaction activity, previous studies in developed countries have shown that social interactions in the neighborhood environment will have considerable health benefits for residents [90]. Among them, studies have shown that in Western low-density developed countries, public transport can improve people’s social interaction levels by replacing car travel [88], which can reduce the risk of obesity and other chronic diseases [56].

Although this study verifies the mediating effect of social interaction activity between the built environment and the health of the elderly in the context of high-density cities in China, it also finds some different conclusions from those in Western developed countries; that is, there is a significant negative correlation between public transport and social networks (Figure 4b). This can be attributed to two reasons. Firstly, in China, the social interaction activity of the elderly around the neighborhood mainly includes chatting, playing cards, and chess [76]. Neighborhoods closed to bus or subway stations, with too many bus and subway stations, will suffer more noise pollution [111]. It will reduce the frequency of social interaction activity (such as chatting, playing cards, and chess), reduce the possibility of informal meetings between the elderly [81,82], and fail to meet the daily social needs of the brain, thereby harming physical and mental health [83]. Secondly, many Chinese elderly people live with their adult children. Just as our questionnaire shows, half of the respondents live with their children. Therefore, they share many family responsibilities, one of which is to pick up their grandchildren to and from school [112]. When the neighborhood where the elderly live is very close to a bus or subway station, and there are many bus and subway stations around, they will choose to rely on public transport [89]. Every time they take public transport to pick up their grandchildren to and from school, it will be within the morning and evening peak hours, which happen to be the most crowded times in the bus or subway [113]. Additionally, this crowded environment is not conducive to the development of social activity and the improvement of physical health [114].

This study still has the following limitations. First of all, our research is based on an analysis of cross-sectional data, which makes it difficult for us to draw a causal relationship between the built environment and the physical health of the elderly. Second, the mediating variables used in this study (physical activity, neighborhood relationship, social network, and neighborhood activity participation) are all indicators that are self-reported by the elderly, and whether there is a relationship between these variables still needs further verification. Additionally, the indicators of physical health used in this study are also derived from subjective questionnaire surveys. If objective indicators close to health were used, such as obesity and body mass index, the research results might be more reliable. Third, considering that we needed to investigate the elderly in all housing types in Guangzhou, it was difficult to collect a large number of elderly questionnaires for a certain type of housing in certain districts, which limited the number of questionnaires we finally collected. Therefore, in future research, we will increase the number of respondents and further enhance the reliability of the research. Finally, the research location selected for this case study is Guangzhou, one of China’s megacities, and the research results need to be further verified for applicability to small and medium-sized cities. Moreover, the rapid and drastic changes in Guangzhou’s built environment have occurred under the stimulus of rapid urbanization and government policies. At the same time, in Chinese culture and consciousness, the Chinese elderly still bear important responsibilities in the family. Therefore, due to the particularity of the development environment, government policies, history and culture, and the limited sample size of this research, the research conclusions cannot be extrapolated to other countries to a certain extent.

## 6. Conclusions

The relationship between the built environment and health has been an emerging but still controversial issue in the fields of urban planning, architecture, and public health. This study used a multilevel mediation effect model, based on the questionnaire survey data of 882 elderly people in 20 neighborhoods in Guangzhou in 2019 and Baidu POI data, to explore the impact of the built environment on the health of the elderly and the mediating path between the two. The research results show that in terms of direct effects, consistent with known conclusions in developed countries, the number of POIs, the distance to the nearest park and square, and the number of parks and squares are significantly positively correlated with the physical health of the elderly. However, contrary to developed countries, the number of bus and subway stations and the distance to the nearest station are significantly negatively correlated with the physical health of the elderly. As for the mediating effect, we found that the availability of parks and squares improves the health of the elderly by promoting physical activity, which is consistent with the research conclusions of Western developed countries. However, unlike Western developed countries, fewer bus and subway stations in neighborhoods will benefit their physical health by expanding their social networks. 

The conclusions of this study not only provide evidence from China regarding theories about the relationship between the built environment and health in the fields of urban planning, architecture, and public health but also have some theoretical reference significance for the planning and construction of healthy neighborhoods, aging-friendly neighborhoods, and a healthy country. 

First, in a city of high-density built environments such as Guangzhou, the diversity of planned land use can be enhanced and the facilities around neighborhoods (such as vegetable and fruit shops, small park squares, and pharmacies) can be made accessible in order to provide opportunities to promote a healthy lifestyle and physical activity for the elderly. Second, it is necessary to increase the number of parks and squares around neighborhoods. Established neighborhoods, especially old and unit housing neighborhoods in city centers, could make full use of corners, abandoned land, and idle land to build pocket parks and small green spaces to meet the needs of the elderly. For neighborhoods planning construction in the future, it will be necessary to reserve public spaces such as parks and squares at the planning stage and include seats, fitness equipment, promenades, and other facilities. Finally, we need to be aware of the negative effects of the number of bus and subway stations and the distance to them in high-density built environments. Therefore, in urban planning and construction in the future, we should reasonably plan and build public transportation stations. On the one hand, accessibility to public transport should be improved to encourage the elderly to use it. It is also important to avoid noise pollution caused by the excessive construction of public transport stations, which requires planners to focus on creating green spaces around them in order to reduce noise and to promote physical activity and social interaction among the elderly.

## Figures and Tables

**Table 1:** Summary statistics for the sampled neighborhoods.

Types of Social Areas	District	Subdistrict	Neighborhood	House Type	Number of Questionnaires Completed
High concentration area of the elderly population in old urban areas	Liwan	Hualin	Xingxian	Historical	23
		Longjin	Huafu	Historical	10
		Lingnan	Yangrendong	Historical	28
	Yuexiu	Zhuguang	Zhujiangyuan	Historical	61
Gathering areas for elderly individuals who have retired from government enterprises and institutions	Liwan	Baihedong	Guangchuanheyuan	Danwei	93
	Haizhu	Nanshitou	Zhibei	Danwei	120
	Huangpu	Huangpu	Huangpuhuayuan	Commercial housing	28
	Tianhe	Yuancun	Meilinhaian	Commercial housing	35
Scattered distribution area of the elderly who retired from educational and scientific research institutions	Tianhe	Wushan	Huagong	Danwei	87
Mixed population distribution area	Liwan	Dongjiao	Fanghehuayuan	Affordable housing	21
	Baiyun	Jinsha	Jinshazhou	Affordable housing	88
	Panyu	Luopu	Guang’ao	Commercial housing	18
	Huangpu	Dasha	Hengsha	Urban village	30
Concentrated distribution area of the rural elderly population	Baiyun	Zhongluotan	Dengtang	Rural village	52
	Baiyun	Zhuyuan	Zhuer	Rural village	25
	Baiyun	Jianggao	Jiangcun	Rural village	20
	Huadu	Huadong	Shanxia	Rural village	47
New development zone with a young population	Baiyun	Xinshi	Tangyong	Urban village	38
	Panyu	dashi	Dashan	Urban village	51
	Tianhe	Tangxia	Tanged	Affordable housing	7

**Table 2:** Descriptive statistics of variables.

Variables	Proportion/Mean	Std	Maximum	Minimum
**Dependent variable**				
Physical health	10.454	2.543	15	3
**Independent variable**				
Population density	1.944	1.824	8.211	0.079
Land use mix	0.667	0.085	0.749	0.454
Number of POIs	4044.568	3546.420	13344	36
Number of parks and squares	4.734	4.519	16	0
Number of bus and subway stations	28.733	16.137	69	1
Distance to nearest park or square (km)	0.482	0.578	2.8	0.016
Distance to nearest bus or subway station (km)	0.267	0.225	0.958	0.040
**Mediating variable**				
Physical activity duration (h)	1.559	1.117	5	1
Neighborhood relationship	3.969	0.660	5	1
Social network	3.732	0.967	5	1
Neighborhood activity participation	1.720	0.739	5	1
**Control variable**				
**Gender**				
Female	56.364%			
Male	43.636%			
**Age**				
60–75	79.205%			
Above 75	20.795%			
**Educational level**				
Primary school and below	40.795%			
Junior middle school	27.614%			
High school or technical secondary school	24.886%			
Training school	4.3182%			
Bachelor’s degree or above	2.386%			
Income	4739.13	4213.04	47500	600
**Lifestyle**				
Live alone or with a spouse	49.659%			
Live with children	50.341%			
**Marital status**				
Unmarried	1.25			
Widowed or divorced	20.682%			
Married	78.068%			
**Individual preferences**				
**Travel model**				
Walk or ride	72.841%			
Public transport	3.75%			
Drive or take taxis	23.409%			
Smoke	17.841%			
Drink	9.205%			

**Table 3:** Associations between the built environment and physical health of the elderly (possible mediators).

	Model 1 DV: Physical Health		Model 2a DV: Physical Activity		Model 2b DV: Neighborhood Relationship		Model 2c DV: Social Network		Model 2d DV: Neighborhood Activity Participation	
	Coef.	SE	Coef.	SE	Coef.	SE	Coef.	SE	Coef.	SE
**Dependent variable**										
**Built environment**										
Population density	−0.024	0.104	−4.158	3.134	0.044	0.027	0.026	0.061	−0.069 **	0.031
Land use mix	2.668	1.829	138.633	58.014	−0.582	0.479	−0.787	1.239	0.772	0.536
Number of POIs	0.0002322 **	0.00009	0.002	0.003	−0.00004	0.00002	−0.00003	0.00006	0.000009	0.00003
Number of parks and squares	0.060 *	0.032	2.766 *	1.002	−0.002	0.008	0.030	0.021	0.019 **	0.009
Number of bus and subway stations	−0.032 **	0.014	0.407	0.255	−0.005	0.004	−0.018 **	0.009	0.003	0.004
Distance to nearest park or square	0.578 **	0.275	−0.622	8.727	0.073	0.072	0.269	0.184	0.230 **	0.081
Distance to nearest bus or subway station	−1.833 **	0.696	16.471	23.008	−0.559 **	0.182	−1.586 **	0.511	−0.650 **	0.204
**Control variable**										
**Socioeconomic attribute**										
Age (ref. 60–75)	−0.516 **	0.221	−14.873 **	5.898	−0.062	0.058	−1.128	0.081	−0.084	0.065
Gender (ref. female)	0.327 *	0.196	3.000	5.237	−0.078	0.051	−0.149 **	0.072	−0.091	0.058
Marital status (ref. unmarried)										
Widowed or divorced	0.624	0.809	−9.680	21.549	−0.225	0.212	−0.049	0.295	0.072	0.237
Married	0.616	0.435	−3.446	21.016	−0.289	0.206	−0.184	0.288	−0.046	0.231
Education level (ref. primary school and below)										
Junior middle school	0.347	0.221	8.754	5.883	−0.047	0.058	−0.035	0.081	0.027	0.065
High school or technical secondary school	0.507 **	0.241	13.251 **	6.439	0.043	0.063	−0.013	0.089	0.040	0.071
Training school	0.218	0.447	13.168	11.916	0.038	0.117	−0.112	0.163	0.168	0.131
Bachelor’s degree or above	0.719	0.587	26.860 *	15.649	−0.274 *	0.154	−0.086	0.214	0.070	0.172
Income	0.322 **	0.093	−4.930 *	2.515	0.051 **	0.024	0.191 ***	0.035	0.047 *	0.027
Lifestyle (ref. live alone)										
Live with children	−0.077	0.175	0.126	4.680	−0.033	0.046	−0.033	0.064	0.013	0.051
**Individual preferences**										
Travel model (ref. walk or ride)										
Public transport	0.034	0.449	6.066	11.975	0.132	0.118	0.348 **	0.164	0.171	0.132
Drive or take taxis	0.083	0.204	0.811	5.450	0.040	0.053	0.112	0.075	0.123 **	0.060
Smoke (ref. no)	−0.238	0.264	−7.670	7.054	0.033	0.069	−0.027	0.097	−0.068	0.077
Drink (ref. no)	−0.205	0.326	5.080	8.692	−0.006	0.085	0.044	0.119	0.117	0.096
Constant	5.679 ***	1.564	31.519	47.466	4.590 ***	0.409	3.681 ***	0.944	0.812 *	0.458
Log likelihood	−2033.375		−4919.8415		−854.6478		−1153.4956		−954.4083	
Prob > chi2	0.0000		0.0136		0.0001		0.0000		0.0000	
AIC	4114.75		9887.683		1757.296		2354.991		1956.817	

Note: ***, **, * significant at the 1%, 5%, and 10% threshold levels, respectively.

**Table 4:** Results of physical health relations: the mediating effect of the four mediators.

	Model 3a Mediator: Physical Activity		Model 3b Mediator: Neighborhood Relationship		Model 3c Mediator: Social Network		Model 3d Mediator: Neighborhood Activity Participation	
	Coef.	SE	Coef.	SE	Coef.	SE	Coef.	SE
**Dependent variable**								
**Built environment**								
Population density	−0.008	0.104	−0.037	0.104	−0.031	0.104	−0.007	0.104
Land use mix	2.114	1.827	2.841	1.826	2.847	1.825	2.487	1.827
Number of POIs	0.0002253 **	0.00009	0.0002429 **	0.00009	0.0002389 **	0.00009	0.00023 **	0.0000892
Number of parks and squares	−0.056 *	0.031	−0.060 **	0.032	−0.069 **	0.032	−0.065 **	0.032
Number of bus and subway stations	−0.034 **	0.014	−0.031 **	0.014	−0.028 *	0.014	−0.033 **	0.014
Distance to nearest park or square	0.581 **	0.274	0.557 **	0.275	0.528 *	0.275	0.524 *	0.276
Distance to nearest bus or subway station	−1.909 **	0.692	−1.668 **	0.697	−1.507 **	0.707	−1.682 **	0.698
**Mediating variable**								
Physical activity	0.004 **	0.001						
neighborhood relationship			0.296 **	0.129				
Social network					0.216 **	0.091		
Neighborhood activity participation							0.233 **	0.115
**Control variable**								
Socioeconomic attribute								
Age (ref. 60–75)	−0.456 **	0.221	−0.497 **	0.221	−0.494 **	0.221	−0.496 **	0.221
Gender (ref. female)	0.317	0.195	0.350 *	0.196	0.356 *	0.196	0.348	0.196
Marital status (ref. unmarried)								
Widowed or divorced	0.665	0.805	0.690	0.807	0.641	0.807	0.607	0.808
Married	0.633	0.785	0.701	0.787	0.661	0.787	0.626	0.787
Education level (ref. primary school and below)								
Junior middle school	0.312	0.220	0.361	0.219	0.355	0.219	0.341	0.220
High school or technical secondary school	0.453 *	0.241	0.494 **	0.240	0.508 **	0.240	0.497 **	0.241
Training school	0.160	0.445	0.207	0.446	0.257	0.446	0.179	0.446
Bachelor’s degree or above	0.608	0.585	0.800	0.586	0.751	0.586	0.703	0.586
Income	0.340 ***	0.093	0.307 **	0.093	0.279 **	0.095	0.311 **	0.093
Lifestyle (ref. live alone or with spouse)								
Live with children	−0.080	0.174	−0.068	0.175	−0.069	0.175	−0.080	0.175
Individual preferences								
Travel model (ref. walk or ride)								
Public transport	0.011	0.447	−0.006	0.448	−0.040	0.449	−0.006	0.449
Drive or take taxis	0.081	0.203	0.071	0.203	0.054	0.204	0.054	0.204
Smoke (ref. no)	0.269	0.263	0.228	0.263	0.245	0.264	0.254	0.264
Drink (ref. no)	0.183	0.325	0.207	0.325	0.198	0.325	0.177	0.326
Constant	5.565 ***	1.556			4.876 **	1.596	5.489	1.563
Log likelihood	−2028.33		−2030.7321		−2030.5733		−2031.3131	
Intra-class variance	0.00000004		1.26 × 10^−6^		8.59 × 10^−7^		0.1475	
Inter-class variance	2.4318		2.4384		2.4379		2.443548	
Prob > chi2	0.0000		0.0000		0.0000		0.0000	
AIC	4106.66		4111.464		4111.147		4112.626	

Note: ***, **, * significant at 1%, 5%, and 10% threshold level, respectively.

**Table 5:** Results of the bootstrap test.

95% Confidence Interval	Physical Activity	Neighborhood Relationship	Social Network	Neighborhood Activity Participation
Number of POIs	(−0.00001, 1.32 × 10^−6^)	(−0.0000187, −2.29 × 10^−8^)	(−0.0000193, −1.91 × 10^−6^)	(−2.93 × 10^−6^, 5.94 × 10^−6^)
Number of parks and squares	(−0.058032, −0.0041877)	(0.0075623, 0.0013637)	(−0.0034038, 0.0041927)	(−0.0001714, 0.0094156)
Number of bus and subway stations	(−0.0013139, 0.000964)	(−0.0029858, 0.0001178)	(−0.003491, −0.0000702)	(−0.0002155, 0.0021805)
Distance to nearest park or square	(−0.0074899, 0.0012913)	(−0.0197871, 0.0237924)	(−0.0491438, 0.0083953)	(−0.0550303, 0.0085365)
Distance to nearest bus or subway station	(−0.1176748, 0.0603461)	(−0.1308616, 0.0274121)	(−0.2816985, −0.0106241)	(−0.2269755, 0.0058005)

## Data Availability

Publicly available datasets were analyzed in this study. These data can be found at http://www.stats.gov.cn/tjsj/tjgb/rkpcgb/qgrkpcgb/ (accessed on 1 June 2021).

## Appendix A. Measurement Scales

**Physical health:**

(i) How do you feel about your health?

(1) Very poor; (2) Poor; (3) Average; (4) Good; (5) Very good.

(ii) Are there restrictions on activities with a large amount of exercise?

(1) Strongly agree; (2) Agree; (3) Generally agree; (4) Disagree; (5) Strongly disagree.

(iii) Have you had physical pain in the past 4 weeks (such as headache, chest tightness, nausea, etc.)?

(1) Strongly agree; (2) Agree; (3) Generally agree; (4) Disagree; (5) Strongly disagree.

**Physical activity:**

How long do you exercise in a day (including walking)?

**________________**(hour)

**Social interaction activity:**

(i) Do you think the relationships in the neighborhood are harmonious?

(1) Strongly disagree; (2) disagree; (3) Generally agree; (4) Agree; (5) Strongly agree.

(ii) Do you know many people in the neighborhood?

(1) Strongly disagree; (2) disagree; (3) Generally agree; (4) Agree; (5) Strongly agree.

(iii) Do you often participate in neighborhood or park activities?

(1) Strongly disagree; (2) disagree; (3) Generally agree; (4) Agree; (5) Strongly agree.
